# Co-design of a novel rehabilitation intervention for patients after ankle fracture surgery: the patient perspective

**DOI:** 10.1007/s12306-025-00887-9

**Published:** 2025-04-02

**Authors:** C. Bretherton, A. Al-Saadawi, H. Sandhu, J. Baird, X. Griffin

**Affiliations:** 1 Bone and Joint Health, Blizard Institute, Queen Mary University London, 4 Newark Street, London, E1 2AT UK; 2 Department of Trauma and Orthopaedic Surgery, Royal London Hospital, Barts Health NHS Trust, London, E1 1BB UK; 3 School of Medicine, Faculty of Medicine and Dentistry, Queen Mary University of London, London, England; 4 Division of Health Sciences, Warwick Clinical Trials Unit, University of Warwick, Coventry, CV4 7AL UK; 5 Centre for Developmental Origins of Health and Disease, University of Southampton, Southampton, SO17 1BJ UK

**Keywords:** Ankle fracture, Rehabilitation, Behaviour Change Wheel, Patient-centred care

## Abstract

**Purpose:**

Effective rehabilitation following ankle fracture surgery is essential for optimal recovery and patient satisfaction. However, traditional rehabilitation strategies often lack personalisation, leading to suboptimal outcomes. This study aimed to co-design a rehabilitation package, collaborating directly with patients, to understand their individual needs, using the Behaviour Change Wheel (BCW) framework.

**Methods:**

Integrated within the larger weight-bearing in ankle fractures (WAX) trial, this study employed the BCW to understand behaviour, intervention options and content implementation. Nominal Group Technique was used to facilitate a workshop with 10 ankle fracture patients, and subsequent surveys were used to gather and prioritise rehabilitation needs and strategies. Participants were aged between 28 and 69, and nine (90%) were female, with representation from seven different NHS hospital trusts. Two experienced patient representatives facilitated the workshop.

**Results:**

Rehabilitation strategies were developed focussing on interventions that included education, training, environmental restructuring, persuasion and enablement, delivered through an app or website. Survey results indicated high patient enthusiasm for structured, accessible rehabilitation support, including instructional videos, live chats with physiotherapists and peer support forums. Patients desired advice on returning to hobbies and life roles, and particularly returning to driving, bathing and work.

**Conclusion:**

The BCW framework facilitated the development of a patient-centred rehabilitation package, highlighting the importance of tailored, accessible interventions. Patients expressed strong support for the proposed strategies, suggesting potential for improved rehabilitation outcomes through personalised, digitally delivered support. These components will be used to co-design future rehabilitation interventions.

## Introduction

### Background

The ankle joint represents one of the most common fracture sites in the lower extremity, affecting 187 per 100,000 people annually [1]. The approach to ankle fracture management depends on joint stability, with unstable fractures necessitating surgical fixation [2, 3]. Rehabilitation is a core component of ankle fracture care, helping restore function following the fracture [4]. To date, rehabilitation interventions revolve around basic movements of the ankle joint, such as ankle dorsiflexion and plantar flexion, and are not specific towards the patient’s individual needs and goals, thereby restricting the maximum potential that can be achieved from the exercise programmes [5]. For instance, Keene et al. suggested that older patients with ankle fractures may benefit more from functional training, such as stair climbing and balance, rather than conventional approaches [6]. Despite increasing evidence supporting early weight-bearing [7], patient adherence to these programmes is a major obstacle, mainly due to anxiety and fear of re-injury [8]. Tailoring rehabilitation towards the patients’ needs has shown to improve their functional capacity in other areas of musculoskeletal care [9, 10].

Co-design or co-production refers to the process of researchers, practitioners and the public working together, sharing power and responsibility to deliver research [11]. Increasing co-production in research has been part of the strategic vision of the National Institute for Health and Care Research (NIHR), primarily to ensure research outputs are relevant and applicable to service users [12]. Various theories and frameworks can be used to design a healthcare intervention. Core tenants for intervention development include using suitable theory, understanding context, establishing key uncertainties and stakeholder engagement [13]. In some cases, existing interventions can be adapted to suit a new context and evaluated. There are no behaviourally based effective interventions in the field of ankle fracture rehabilitation [5]. Furthermore, there is significant uncertainty around the beliefs and behaviours of patients and healthcare professionals (HCPs) with respect to weight-bearing [14]. Taking this into account, adapting an existing intervention is not suitable.

The Behaviour Change Wheel (BCW) is a framework that aids in the design and evaluation of interventions through the application of 19 distinct behavioural models [15]. The BCW is ideally placed to co-design an intervention de novo [12, 13]. It begins by deconstructing the problem or need, defining it in its base behavioural components and follows a systematic process for addressing the need. The three core phases of the BCW are: (1) understand the behaviour, (2) understand the intervention options and (3) understand the content and implementation options. Once the behavioural problem has been identified, the most common methodology used in phases two and three of the BCW is a qualitative approach in which interviews and/or workshops are used to gain in depth data to help define behavioural problems and develop solutions with stakeholders [16–18]. Roberts et al. combined qualitative and quantitative approaches to identify significant barriers and facilitators to implementing a Sepsis Six care bundle in hospitals. They used analysis of semi-structured interviews to generate belief statements which informed a questionnaire to determine the generalisability of belief statements among a wider group of stakeholders [19]. This study follows a similar approach but adds in an intermediate step of the generation of behavioural intervention bundles to facilitate discussion at stakeholder workshops, as proposed by Shallcross et al. [20]. The objectives of this study were to determine intervention options for a rehabilitation package after ankle fracture surgery and identify content and implementation options through consultation with patients.

## Methods

### Study design

The study was incorporated within a mixed methods study, including the large-scale weight-bearing in ankle fractures (WAX) trial and qualitative interview studies, published separately [7, 8, 21]. WAX was a multicentre, randomised controlled trial conducted in the UK that aimed to study early weight-bearing following operatively managed ankle fractures compared to traditional delayed weight-bearing regimens. A protocol for the WAX trial was registered on the 2nd of December 2019 (ISRCTN12883981) and has been published [22]. The South Central Oxford A Research Committee granted ethical approval for this study on the 22nd of November 2019 (Reference: 19/SC/0566).

A rehabilitation strategy was co-designed using the three phases of the BCW [15]. Phase one of the BCW, "Understand the behaviour" was done by assessing participants’ capability, opportunity and motivation to engage with the behaviour (weight-bearing) in prior studies within the WAX trial. Phases two and three are the subject of this paper.

#### Understand the intervention options

Key barriers, enablers and areas needing change as established in prior studies within the WAX trial were mapped onto the nine intervention functions of the BCW [23]. A list of candidate behaviour change intervention bundles was generated [20] using analysis methods described in the analysis section below.

#### Understand the content and implementation options

**Fig. 1:**
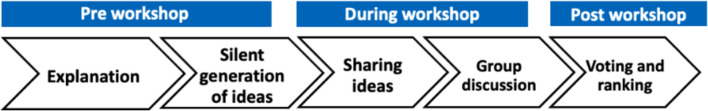
Online Nominal Group Technique (NGT)

This was done using the Affordability, Practicability, Effectiveness/Cost-effectiveness, Acceptability, Safety/Side effects (APEASE) criteria [15]. The formulated behaviour change intervention bundles were presented and discussed at a workshop attended by 10 patients recovering from ankle fracture surgery. All participants were then sent a survey, asking them to rank the importance of different aspects of the intervention bundles and content. An online Nominal Group Technique (NGT) (Fig. 1) was used for the patient workshop and survey, reported in accordance with the Checklist for Reporting of Survey Studies (CROSS) [24].

#### Setting and sampling

All patients were recruited through the WAX trial, with inclusion and exclusion criteria reflecting the WAX trial criteria [7]. During recruitment to the WAX trial, participants were asked to indicate if they were happy to be contacted for a qualitative interview or discussion workshops (this included patients who declined enrolment into the WAX trial). A maximum variation sample of patients from different geographical, demographic and socio-economic backgrounds at different stages in recovery from their ankle fracture was recruited. Participants were offered a £75 shopping voucher as an incentive to participate, as recommended by our patient representatives and approved by the ethics committee (Reference: 19/SC/0566). Patients were provided with a Participant Information Sheet specific to the workshops. The workshop was conducted virtually, and patients completed an online consent form before the workshop.

#### Data collection methods

An online Nominal Group Technique (NGT), as developed and described by Bateman et al. [25] and Fisher et al. [26], was used. The workshop took place between 10 am and 4 pm on 13th December 2021. Participants were split into three breakout groups, facilitated by the lead author (CB) and two experienced patient representatives. Assignment of breakout groups was done to maximise demographic and time-from-injury diversity within groups to facilitate a discussion of a wide range of rehabilitation needs at different time points.

All participants completed pre-workshop tasks, including watching an explanatory video and completing a worksheet (Appendix A1). The worksheet included separate sections for different intervention bundles. Participants were presented with an illustrative quote from patient and healthcare professional interviews conducted in previous studies part of the larger WAX trial and asked to consider if they could relate or identify with the quote which expressed a particular concern, struggle or need. They were then asked to consider what additional content would be helpful to address their concern or need and how they would like that support or content delivered. Additionally, they were asked to consider any potential drawbacks of this new service and if any particular groups of patients might be excluded or find the new service challenging to engage with.

During the workshop, participants progressed through the worksheet discussing intervention bundles corresponding to different COM-B (Capability, Opportunity, Motivation—Behaviour) domains. All breakout groups were recorded. After the workshop, all recordings were reviewed, and notes from facilitators were compiled to create content for the survey.

#### Survey

An online survey was compiled using data from the workshop. The online survey link was emailed to participants and completed within two weeks of the workshop. Participants were asked to indicate how helpful various advice and support would be for their recovery by ranking statements on a five-point Likert scale from "very unhelpful" to "very helpful".

### Analysis

#### Generation of intervention bundles

Intervention bundles were generated by mapping target themes/belief statements generated during prior studies within the WAX trial to intervention functions from the Behaviour Change Wheel (BCW) [15]. "Intervention functions" relate to a range of methods that can be used to change behaviour. NICE first issued the principles for selecting and evaluating behaviour change interventions [27]. Later, Michie et al. conducted a systematic review of intervention functions and produced mapping matrices to link COM-B domains to intervention functions that were most likely to bring about behaviour change [23].

Once target intervention functions were identified, these were mapped onto potential policy categories using the relevant matrices [15]. After establishing potential intervention functions and policy options, the corresponding behaviour change techniques (BCTs) were selected from the BCT taxonomy [28]. The taxonomy and a complete list of potential BCTs can be found in Appendix A2. BCTs were chosen using the BCW guide, which lists the most suitable and commonly used BCTs for each of the nine possible intervention functions [15].

The resulting target behaviour, intervention function, policy category and corresponding BCT formed an "Intervention bundle", which was then presented to stakeholders during the workshop. For simplicity, no reference to behaviour change theory was used during the workshop. Instead, the intervention bundles were broken down into relatable concepts and categories as demonstrated in Appendix A1.

#### Analysis of survey responses

Analysis was conducted in R. Participant characteristics were summarised as frequencies and percentages. Survey figures were compiled using the package Likert [29, 30]. The reporting of figure summaries was grouped according to COM-B domains. Seventy-five per cent was used as a threshold for consensus [31, 32].

## Results

### Intervention bundles

Table 1 shows the list of intervention bundles used for this study. The intervention functions selected were "Education", "Training", "Environmental Restructuring", "Persuasion" and "Enablement". The policy category selected to modify patients’ behaviour was "Service provision", and to modify HCPs behaviour, "Guidelines" was selected. Corresponding BCTs are listed in the last column of Table 1.

**Table 1:** COM-B intervention bundles

COM-B/TDF domains	Intervention functions	Policy options	Behaviour change techniques
**Capability—Physical** **2. Skills** Uncertainty how to weight-bear or use crutches Struggling to perform ADLs	**Training** How to use walking aids and progress weight-bearing How to perform ankle exercises How to perform ADLs	**Service Provision** An App/website with instructional videos Live chat to a physiotherapist	4.1 Instruction on how to perform the behaviour 6.1 Demonstration of the behaviour 8.1 Behavioural practise/rehearsal 8.7 Graded tasks
**Capability—Psychological** **2. Knowledge** What’s normal? What to expect? **10. Memory, attention and decision processes** The perception that exercises only happen during physiotherapy clinic Forgetting to do exercises	**Education** Timeframes for recovery, personalised to the patient Pictures of normal and concerning wounds **Environmental restructuring** Reminders to do physiotherapy	**Service Provision** An App/website with explanations Live chat to a physiotherapist Collect information from patients about their recovery which can then be used to update the App timeframes information Exercise reminders via an App, with or without a diary	5.1 Information about health consequences 7.1 Prompts/Cues 2.3 Self-monitoring of behaviour 7.1 Prompts/Cues 2.3 Self-monitoring of behaviour
**Opportunity—Physical** **11. Environment** Lack of access to physiotherapists Lack of information between appointments Uncertain what aids would help or where to get them	**Training** Which resources to use and how Environmental restructuring Alternative access to HCPs (i.e. virtual)	**Service Provision** An interactive website or App A national physiotherapist on-call 9-5 pm to answer questions via the App or videoconference	9.1. Credible source 1.4. Action planning
**Opportunity—Social** **12. Social influences** Peer support: sharing advice and experiences Advice for family and friends	**Environmental restructuring** Group physiotherapy A method for providing peer support **Education** Education resources for family and friends	**Service Provision** Virtual group physiotherapy classes A Peer support forum via an App	3.1 Social support (unspecified) 3.2. Social support (practical) 6.2. Social comparison
**Motivation—Reflective** **4. Beliefs about capabilities** Improve self-efficacy Knowledge/beliefs of benefits and lack of harm of walking and exercising Reduce conflicting HCP advice **9. Goals** Establish patient-specific goals	**Persuasion** Persuade patients that they have the capability to walk/exercise **Education** What to expect during their recovery Help patients identify red flag signs **Enablement** Set goals and develop strategies for coping with pain	**Service Provision** An App/website with instructional videos. Live chat to a physiotherapist or virtual appointments **Guidelines** Establish consensus for rehabilitation pathways and advice	15.1. Verbal persuasion about capability 5.1. Information about health consequences 1.1. Goal setting (behaviour)
**Motivation—Automatic** **13. Emotion** Pain avoidance and fear of weight-bearing	**Enablement** Set goals and develop strategies for coping with pain **Education** What to expect during their recovery/what’s normal	**Service Provision** An App/website with instructional videos Live chat to a physiotherapist	9.1. Credible source 15.1. Verbal persuasion about capability 5.1. Information about health consequences

TDF: Theoretical Domains Framework, HCP: Healthcare professionals, ADL: Activities of daily living

### Participants

Fifty participants were invited to participate in the workshop, of which 10 (20%) agreed and attended. Participants were aged between 28 and 69, and nine (90%) were female, with representation from seven different NHS hospital trusts. Participant sites and characteristics can be seen in Tables 2 and 3, respectively, in Appendix A3.

All participants completed the pre-workshop tasks (confirmed during the workshop initiation) and post-workshop survey. In addition, the two patient representatives also completed the survey based on their personal experience of injury or from caring for relatives with an ankle injury.

### Survey results

**Fig. 2:**
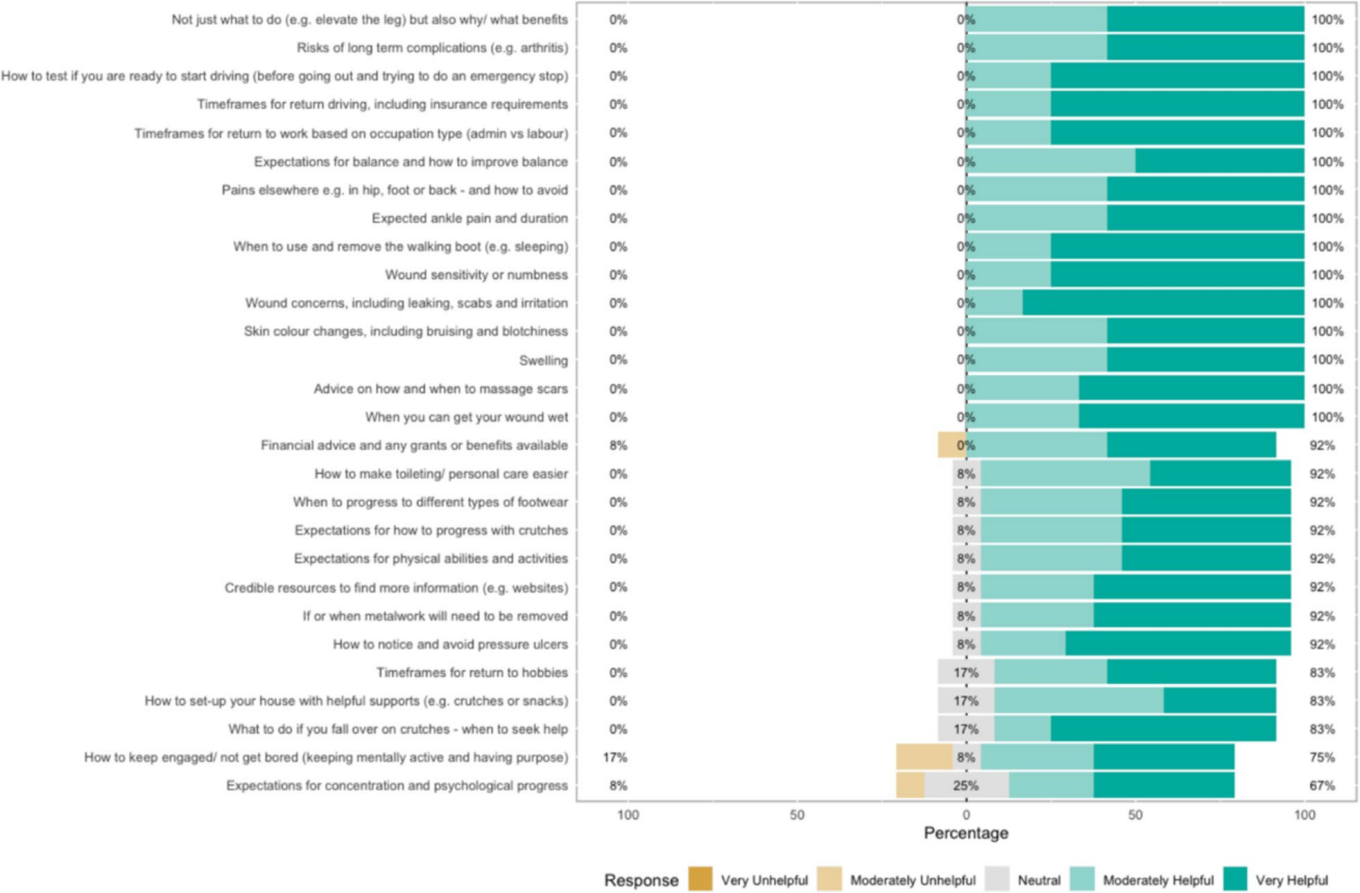
Patient survey—knowledge

**Fig. 3:**
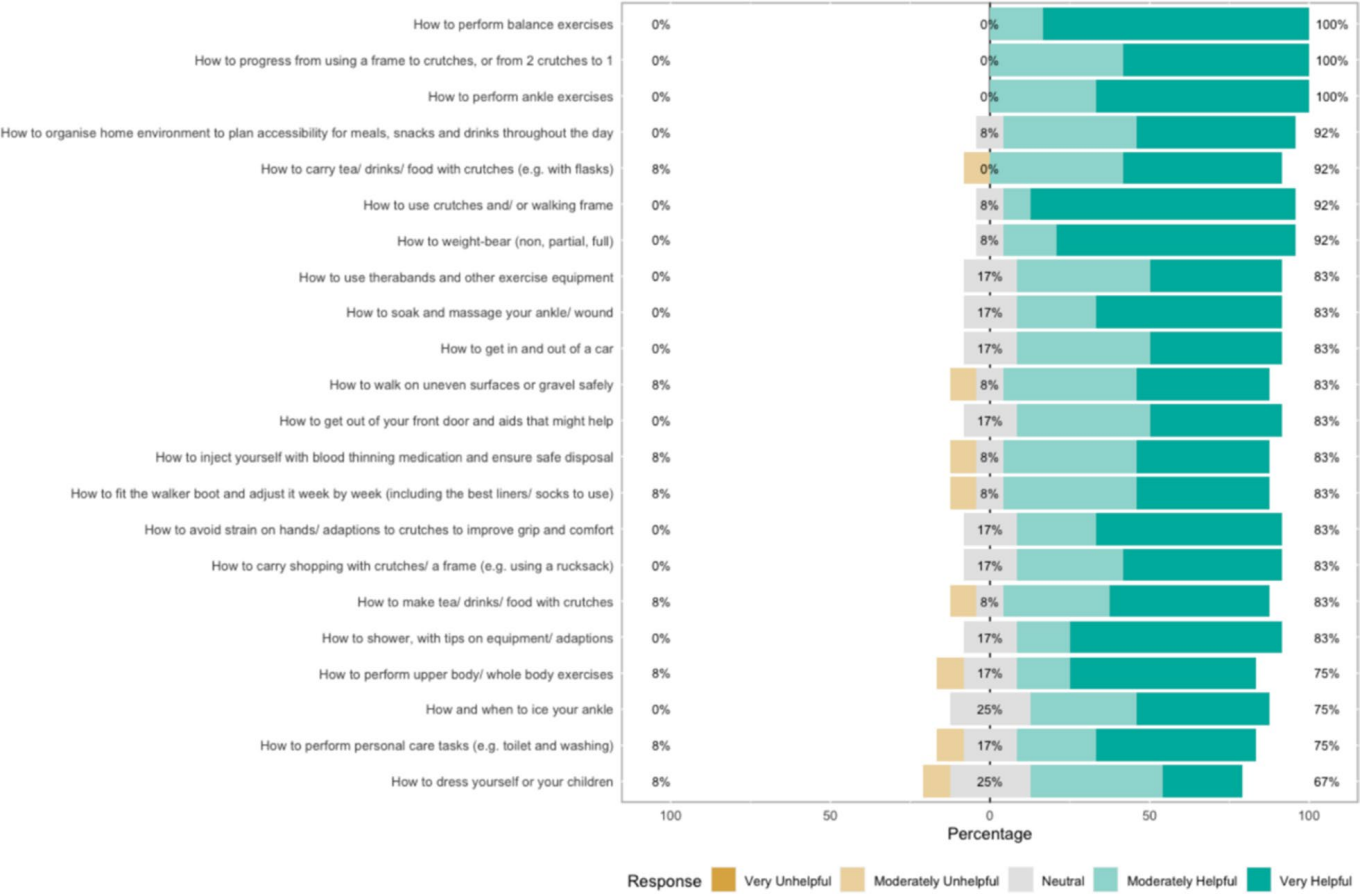
Patient survey—skills

**Fig. 4:**
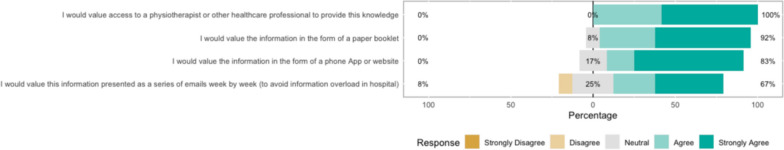
Patient survey—knowledge delivery

**Fig. 5:**
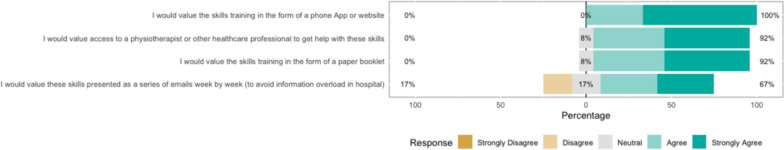
Patient survey—skills delivery

**Fig. 6:**
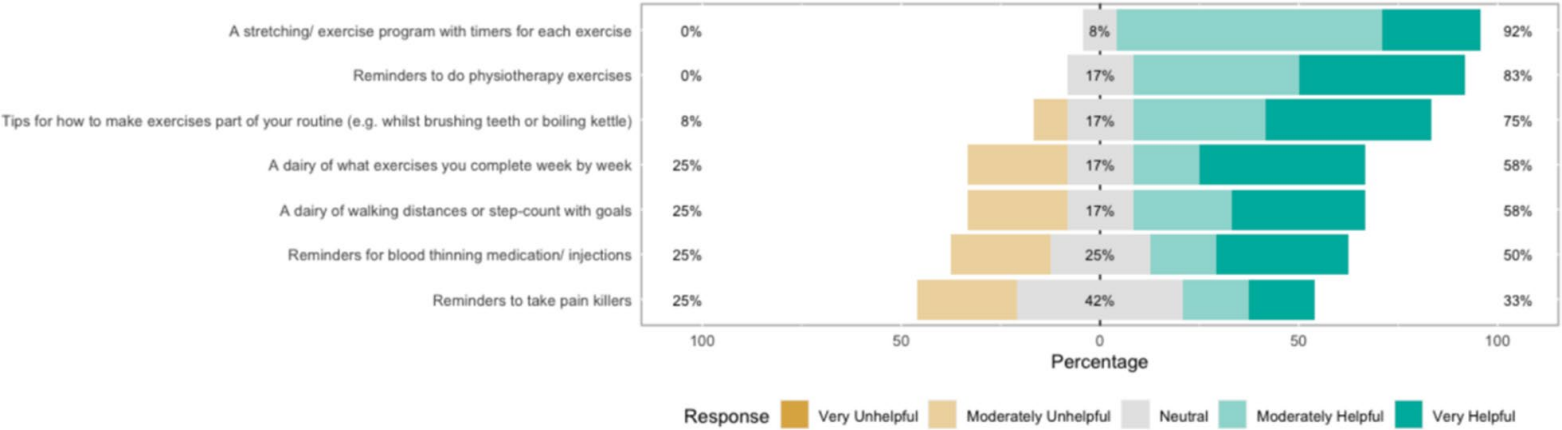
Patient survey—reminders

**Fig. 7:**
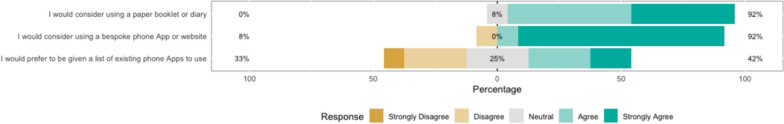
Patient survey—reminders delivery

**Fig. 8:**
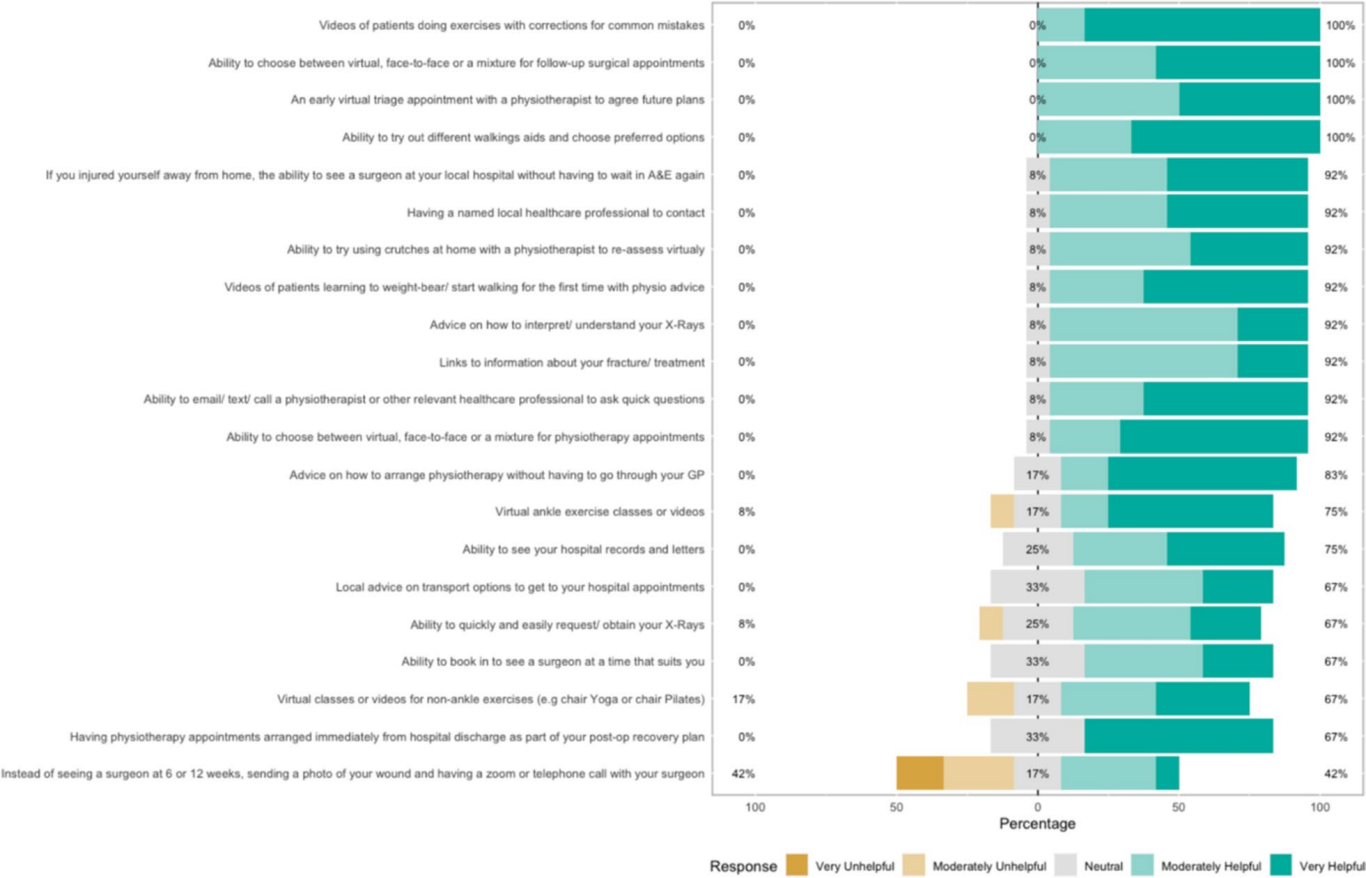
Patient survey—HCP access

**Fig. 9:**
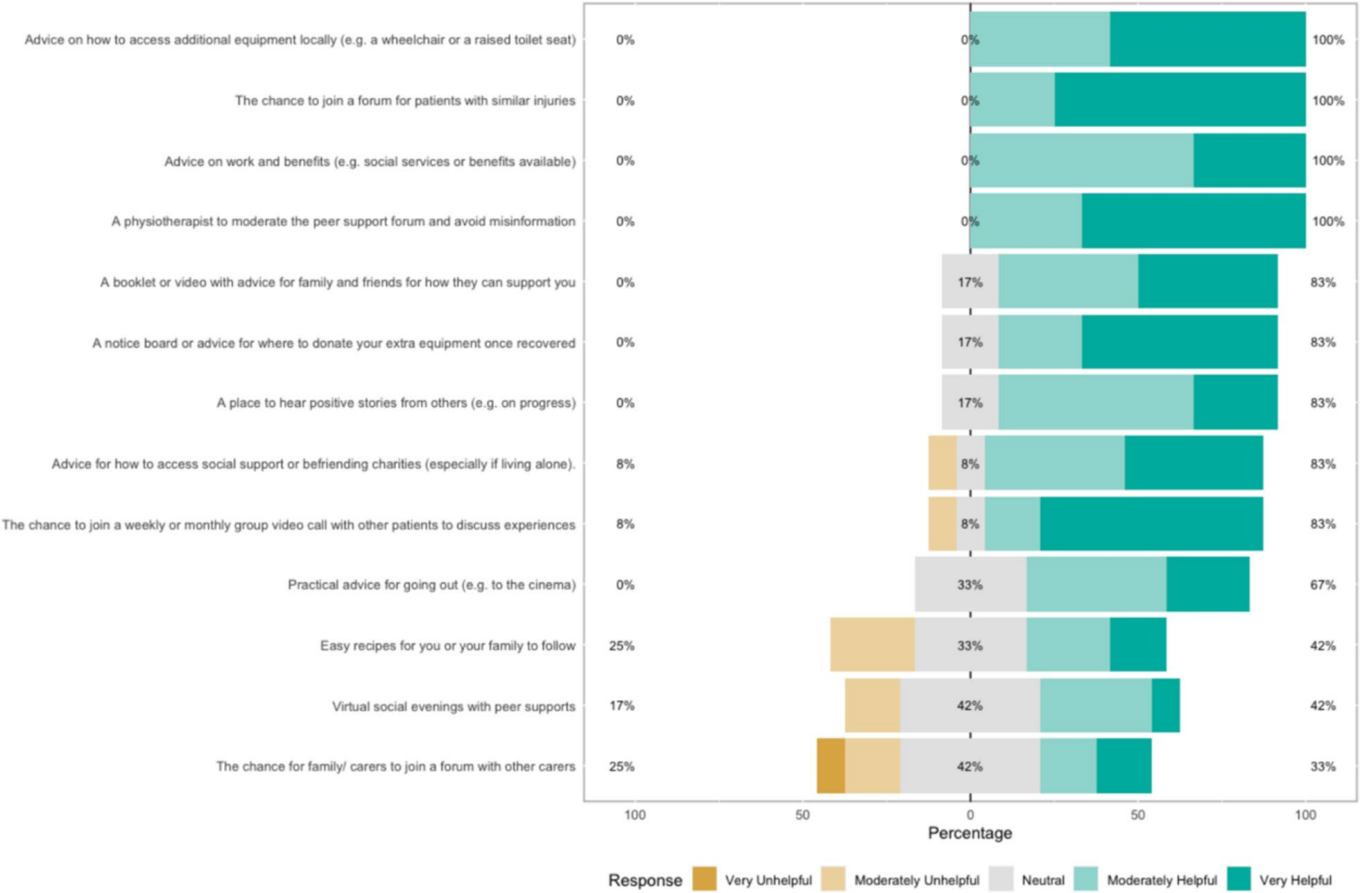
Patient survey—social support

**Fig. 10:**
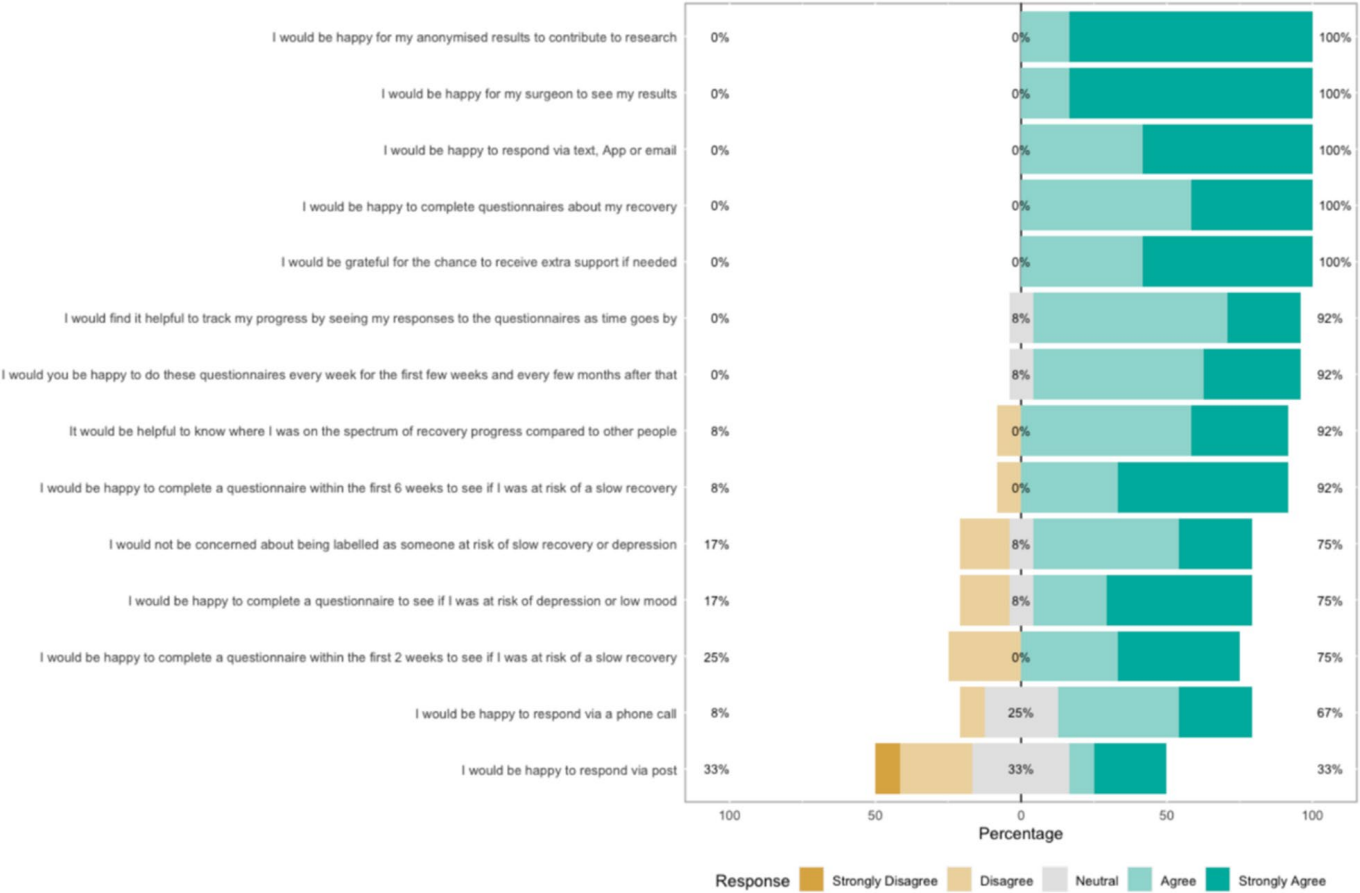
Patient survey—monitoring

**Fig. 11:**
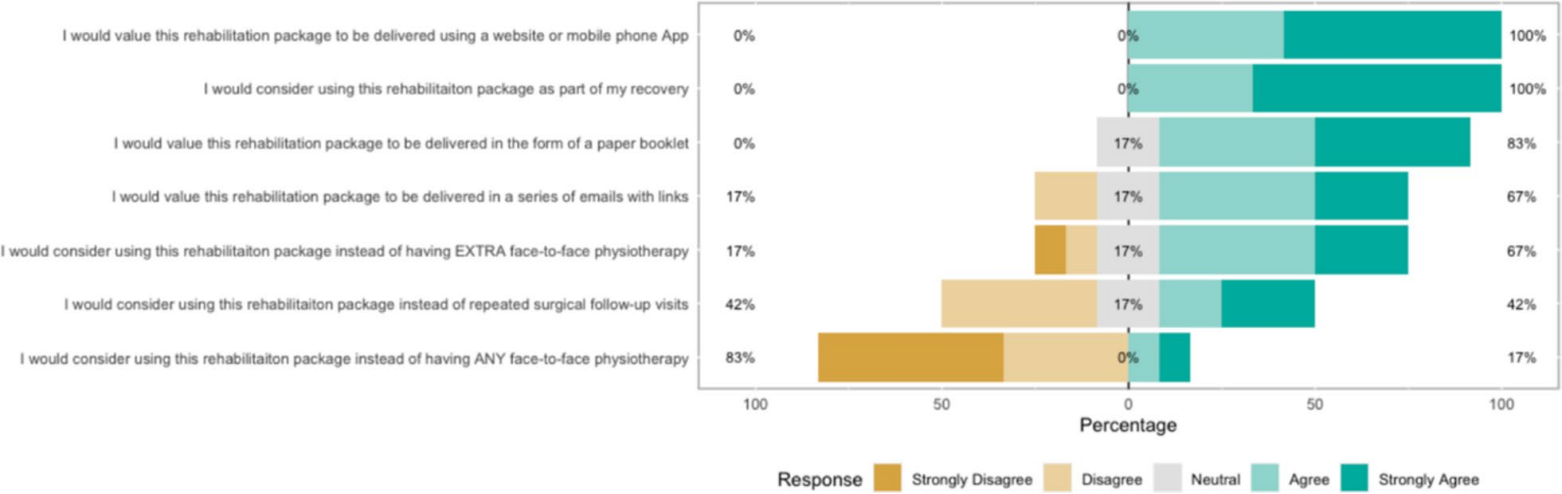
Patient survey—overall delivery

The survey results show how strongly patients agreed with a variety of statements. The Likert bar plots are displayed in Figs. 2, 3, 4, 5, 6, 7, 8, 9, 10 and 11. The percentages on the right side of the Likert bar plots correspond to the proportion of responses that were ranked "moderately helpful" or "very helpful", or "agree" or "strongly agree". The percentages on the left correspond to those ranked "moderately unhelpful" or "very unhelpful", or "disagree" or "strongly disagree", and the middle grey percentages to "neutral". Though 75% was stated as the level for consensus, in many cases regarding the content of a novel rehabilitation service, it was not the absolute percentage that was important, but instead it gives an indication of which content should be prioritised in future stages of development.

#### Knowledge

Figure 2 shows the survey results within the Knowledge domain. Physical concerns included advice around swelling, skin colour changes and wound management. Providing expectations was also important, including time frames for returning to driving, work and hobbies, as well as expectations for a full recovery and the likelihood of suffering long-term functional restrictions or complications. There was also uncertainty about if and when patients would need removal of their ankle metalwork, and consistency of advice was desired. Helpful additional advice desired included advice on available financial support, how to optimise the home layout and when to use or discontinue using their post-operative boot and other resources. There was less enthusiasm for general psychological support, but all felt the need for credible information resources.

#### Skills

Figure 3 shows the survey results within the Skills domain. Patients responded with the desire for additional skills training around weight-bearing, exercise and activities of daily living. They wanted skills training for mobilising on crutches and progressing with weight-bearing towards independence. Advice around ankle exercises, the use of exercise equipment, and how to perform non-ankle (e.g. upper body) exercises to keep fit was desired.

Several patients mentioned wound massage during the workshops; however, many were uncertain when and how to perform this and desired additional skills training in wound management. Patients also wanted extra advice on adjusting their orthotic boot and using injectable VTE prophylaxis.

Advice was desired around life skills that were particularly challenging while non-weight-bearing or using crutches, such as cooking, carrying drinks and showering.

#### Knowledge and skills delivery

Figures 4 and 5 show the results of the survey regarding the delivery of the Knowledge and Skills domains. There were similar preferences for the delivery of advice. Most were open to receiving online advice, advice directly from a HCP or from a paper booklet. However, patients were less enthusiastic about receiving a series of emails week after week and seemed to prefer the ability to dip into information at their own pace, as well as seeing what was to come in their recovery.

#### Reminders

Figures 6 and 7 show the survey results for the Reminders and Reminders delivery domain. Most patients would have liked a service that provided them with reminders to perform physiotherapy exercises and felt a structured programme with inbuilt reminders would be helpful, as well as tips for building this into their daily routine.

Patients were less interested in using an exercise programme for keeping a diary and tracking their progress, feeling that this might overburden them with tasks. Most patients preferred such a reminder service to be delivered in the form of a booklet or a bespoke rehabilitation App rather than being given a generic list of existing Apps.

#### HCP access

This section considered novel methods of interacting with HCPs. Figure 8 shows the survey results within this domain. Regarding the organisation of care, there was a desire for the ability to make clinic bookings more easily and flexibly. Additionally, some patients expressed frustration with having to wait in Accident and Emergency department (A&E) multiple times if injured away from their home region, and the majority agreed being referred directly to their local orthopaedic service would be preferable.

With regard to physiotherapy, most would have liked earlier physiotherapy contact as an outpatient. Proposed helpful physiotherapy functions included videos of patients doing exercises or using different walking aids with feedback or virtual physiotherapy appointments to get direction and feedback. Several patients were interested in virtual group therapy classes, including yoga and pilates. Patients were enthusiastic about the ability to have a live messenger chat with a physiotherapist to answer questions, particularly if that physiotherapist had access to their relevant medical records. There was (mixed) interest in the ability to avoid additional surgical follow-up by instead sending pictures of their wounds and receiving virtual advice.

#### Social support

Figure 9 shows the survey results within the Social support domain. Advice on where to procure additional rehabilitation equipment or a sharing message board was discussed, particularly as patients usually only needed equipment for short periods of time and did not want to waste it. This concept of patients sharing information, experiences and advice was also seen as very helpful. Many found that even the opportunity to discuss with other people in similar situations with similar injuries during the patient workshop was valuable. Suggestions were that this peer support could be organised in the form of a chatroom or virtual events. There was a consensus that having a HCP to moderate a forum and avoid misinformation would likely be useful.

#### Monitoring

Figure 10 shows the survey results within the Monitoring domain. The idea of remotely monitoring patients’ recovery was discussed. This was primarily to allow data to be used for research or surgeon feedback, and as long as this was secure and anonymised, all were happy with this. Patients were amenable to responding to questionnaires or providing this information via a text link on their phone or via email, as opposed to post or phone. Having weekly questionnaires in the early stages and tapering off thereafter was considered reasonable.

Patients also felt this function could be used to track their progress and motivate themselves and that the data could be used to identify patients that were recovering slowly or had poor scores, particularly if they could then be flagged for additional support or assessment. They could also compare themselves to other people with similar injuries. The idea of screening patients that were at risk of depression or slow recovery (e.g. those with unhelpful pain beliefs) was discussed, and patients were amenable to this, but there was debate as to when this was best done: two weeks was considered too early by some, but most thought the six-week time point would be reasonable.

#### Overall delivery

Figure 11 shows the results of the survey with regard to the overall delivery of the rehabilitation package. During the workshop and survey, patients were asked about their preferences for the overall delivery of the proposed additional support. Most felt it would be a valuable addition to their recovery that they would engage with, and that a website or App would be the ideal delivery method. Patients had mixed views about the usefulness of their six-week surgical follow-up visit and felt they would consider forgoing this appointment if they had access to the proposed rehabilitation App. Similarly, they would consider having less F2F physiotherapy if the virtual option was available. However, there was a consensus that even with the App, patients would still want to see a physiotherapist F2F at least once during their recovery.

## Discussion

### Interpretation

The Behaviour Change Wheel (BCW) has previously been used to systemically generate behaviour change intervention bundles for a range of health conditions, from sexual counselling to antibiotic stewardship [20, 33]. This is the first attempt to use the method in orthopaedic trauma. A consensus of patients was enthusiastic about using the proposed rehabilitation package, with the strongest opinions suggesting it should be delivered in the form of a website or App. The following presents key areas that require the generation of consensus advice from HCPs or consideration of how the bundles would be ideally delivered within the context of the NHS [31–33].

Patients desired a range of information about their recovery, from knowledge about how to interpret their symptoms to when they should return to work. Patients would like information on their risk of complications and expected time frames for return to life roles. While individual research articles present rates of complications and mean Patient-Reported Outcome Measures (PROMs) at certain time periods, this is not in a risk-stratified or accessible format [34, 35]. A systematic approach for gathering and presenting these data would need to be developed, but first, a consensus for its importance among HCPs would need to be established to ensure engagement with the data collection process. There is literature which provides likely time frames for returning to driving, though Burnham et al. showed that patients often returned to driving sooner, not based on advice or time frames, but when they felt they were ready based on their self-assessment [36, 37]. A consensus around when or how HCPs advise people to return to driving will enable harmonisation of advice with recommendations from the literature.

Randomised controlled trials and systematic reviews have been published comparing early vs delayed wound washing after surgery, with low-quality evidence suggesting that washing within 48 h may be safe [38, 39]. However, these studies have been in heterogeneous populations, primarily for minor procedures without retained metalwork. Considering the anxiety expressed by HCPs regarding variable post-operative advice, establishing a consensus opinion to guide patients is likely more suitable. Similarly, advice on when to use and remove the post-operative orthotic boot was variable and would require HCP consensus before providing standardised advice.

Patients were open to alternative methods of clinical follow-up and were enthusiastic about being able to contact a physiotherapist as and when needed to discuss anxieties and concerns in real-time. Agreement on what symptoms patients can be reassured of, and which symptoms require further assessment must be established. Furthermore, the feasibility and limitations of assessing patients virtually need consideration, as challenges including access to technology and altered patient engagement have been highlighted previously [40]. A consensus among HCPs is required for when patients could be discharged from routine surgical follow-up to the proposed App. Additionally, the required minimum clinical information and notes for a physiotherapist delivering this service need to be defined.

Patients felt it would be acceptable to screen for unhelpful pain beliefs or other pre-existing characteristics that might predispose them to a difficult or slower recovery. There was interest from patients regarding mechanisms for enabling and promoting peer support. While peer support forums can facilitate shared advice and empowerment, with unmoderated forums there is a significant risk of misinformation spreading among patients, which can ultimately foster negative outcomes [41]. Therefore, the acceptability of psychological screening and peer support to Health care professionals needs to be established and the optimal method for when, where and how this should take place is unclear and requires further investigation.

### Strength and limitations

The use of the BCW to generate the template intervention bundles ensured that the proposed intervention and policy options were informed by relevant theory, based on techniques that have been effective in prior research [15, 42]. This method facilitated a focussed discussion at the workshop and ensured the outputs were aligned with congruent theory. The systematic and transparent nature of the BCW method used also ensures that researchers will be able to trace the interventions to their underlying BCTs for faithful replication or evaluation [13].

The design, facilitation and analysis of the patient workshops by patient representatives resulted in a wider range of interpretation and less individual-researcher bias resulted. Ten patients may not have provided a representative sample for the consensus, although this is a relatively large sample for the design of BCW-based intervention compared to previously published studies [16, 18]. However, 90% of workshop participants were female, half of whom were retired or not in employment. The under-representation of men in behavioural research has been reported elsewhere [43]. While efforts were made to increase the participation rate and include more younger and male participants (50 invites were sent and a £75 shopping voucher incentive was offered), most were unable to attend an all-day workshop during working hours. This selection bias may limit the generalisability of the findings, representing only one-half of the bimodal distribution of ankle fractures but not the other half, which occur in young men [44]. This is important, as younger, particular self-employed men have been shown to have poorer outcome after ankle fractures and so it is crucial that future research seeks to understand if this is due to systemic deficiencies in the rehabilitation support they receive [45].

The next steps in the research include achieving a consensus among healthcare professionals around the topics and themes established to help decrease conflicting advice and empower patients to progress their recovery independently. While Behaviour Change Wheel methodology has been used widely for intervention design, adaptions may be required for this orthopaedic application. Once established, the intervention would require evaluation in a clinical setting, likely with an initial feasibility study to evaluate the acceptability, sustainability and applicability to a broader patient population [46].

## Conclusion

This paper has prioritised the intervention methods and content for facilitating weight-bearing and rehabilitation after ankle fracture surgery. Intervention bundles have been created to address the barriers and harness the facilitators in implementing early weight-bearing. Patients have shown openness to engaging with HCPs through non-conventional methods, and the knowledge and skills required for patients to self-manage their recovery have been established.

## Appendix

### Appendix A2

Labels of the BCTs within the taxonomy [15]

### Appendix A3

See Tables 2 and 3.

**Table 2:** Workshop participant sites (patients)

Site	*N* = 10
Royal United Hospitals Bath	3 (30%)
Dorset County Hospital	1 (10%)
East Surrey Hospital	1 (10%)
Maidstone and Tunbridge Wells Hospital	1 (10%)
Oxford University Hospitals	1 (10%)
Queen’s Medical Centre Nottingham	1 (10%)
Royal Preston Hospital	1 (10%)

**Table 3:** Workshop participant characteristics (patients)

Participant ID	WAX Participant?	Interview Participant?	Age	Gender	PESQ-2 Score	Occupation	Days post-surgery
1	Yes	Yes	68	Female	4	Not employed	662
2	Yes	Yes	65	Female	12	Not employed	192
3	Yes	Yes	69	Female	5	Not employed	175
4	Yes	Yes	43	Female	4	Not employed	161
5	Yes	Yes	68	Female	10	Not employed	159
6	Yes	No	28	Female	2	Sedentary	105
7	Yes	No	45	Female	6	Intermediate	103
8	Yes	No	55	Female	10	Sedentary	88
9	Yes	No	40	Female	8	Sedentary	80
10	No	No	44	Male	NA	Labour	14
